# Ciliary neurotrophic factor is increased in the plasma of patients with obesity and its levels correlate with diabetes and inflammation indices

**DOI:** 10.1038/s41598-022-11942-x

**Published:** 2022-05-18

**Authors:** Jessica Perugini, Eleonora Di Mercurio, Angelica Giuliani, Jacopo Sabbatinelli, Anna Rita Bonfigli, Elena Tortato, Ilenia Severi, Saverio Cinti, Fabiola Olivieri, Carel W. le Roux, Rosaria Gesuita, Antonio Giordano

**Affiliations:** 1 Department of Experimental and Clinical Medicine, School of Medicine, Marche Polytechnic University, Via Tronto 10/A, 60020 Ancona, Italy; 2 Department of Clinical and Molecular Sciences, Marche Polytechnic University, Ancona, Italy; 3 Laboratory Medicine Unit, United Hospitals, Ancona, Italy; 4 Scientific Direction, IRCCS INRCA, Ancona, Italy; 5 Diabetology Unit, IRCCS INRCA, Ancona, Italy; 6 Center of Obesity, Marche Polytechnic University-United Hospitals, Ancona, Italy; 7 Center of Clinical Pathology and Innovative Therapy, IRCCS INRCA, Ancona, Italy; 8 Diabetes Complications Research Centre, Conway Institute, School of Medicine, University College Dublin, Dublin, Ireland; 9 Center of Epidemiology and Biostatistics, Marche Polytechnic University, Ancona, Italy

**Keywords:** Diabetes, Metabolic syndrome, Obesity, Endocrinology

## Abstract

To establish whether obesity involves activation of endogenous ciliary neurotrophic factor (CNTF) signalling, we evaluated its plasma levels in patients with obesity and correlated its values with the major clinical and haematological indices of obesity, insulin resistance and systemic inflammation. This study involved 118 subjects: 39 healthy controls (19 men), 39 subjects with obesity (19 men) and 40 subjects with obesity and diabetes (20 men). Plasma CNTF and CNTF receptor α (CNTFRα) were measured using commercial ELISA kits. The results showed that plasma CNTF was significantly higher in males and females with obesity with and without diabetes than in healthy subjects. Women consistently exhibited higher levels of circulating CNTF. In both genders, CNTF levels correlated significantly and positively with obesity (BMI, WHR, leptin), diabetes (fasting insulin, HOMA index and HbA1c) and inflammation (IL-6 and hsCRP) indices. Circulating CNTFRα and the CNTF/CNTFRα molar ratio tended to be higher in the patient groups than in controls. In conclusion, endogenous CNTF signalling is activated in human obesity and may help counteract some adverse effects of obesity. Studies involving a higher number of selected patients may reveal circulating CNTF and/or CNTFRα as potential novel diagnostic and/or prognostic markers of obesity, diabetes and associated diseases.

## Introduction

Ciliary neurotrophic factor (CNTF) belongs to the interleukin (IL)-6 cytokine family along with IL-11, oncostatin M, leukaemia inhibitory factor (LIF), cardiotrophin (CT)-1 and CT-like cytokine factor 1^1,2^. Rather than sequence homology, these cytokines share a four-α helical bundle structure containing discrete motifs responsible for binding high-affinity α receptors on target cells. Binding of CNTF to CNTF receptor α (CNTFRα) is followed by interaction with LIF receptor β (LIFRβ) on target cells and by activation of the widely distributed signal transducing protein gp130^2,3^. Originally identified for its role in supporting the survival of chick ganglion neurons during development in vitro^4^, CNTF has subsequently been shown to exhibit multiple and pleiotropic effects during nerve tissue development, including proliferation of neural progenitors, differentiation of sympathetic neurons, maturation of glial progenitor cells into astrocytes and oligodendrocytes and postnatal maintenance of sensory and motor neurons^5,6^.

Studies performed in experimental animals have found that CNTF is produced by a limited number of cell types in the body, including astrocytes and tanycytes in the brain^7–10^, Schwann cells in peripheral nerves^7,11^ and bone cells^12^. Interestingly, the *cntf* gene lacks a sequence coding for a peptide signal capable of directing the synthesis and secretion of the protein via the classic endoplasmic reticulum-Golgi pathway, and there is evidence that CNTF is a cytosolic protein^11^. These observations, together with the rapid upregulation of the *cntf* gene, seen in grey matter astrocytes after lesion or deafferentation^8,13,14^ and in sciatic nerve Schwann cells in response to lesion^15,16^, suggest a role for CNTF as a protective factor which becomes available after cell injury, death or other types of stress to repair target cells through paracrine and/or endocrine signalling. In this context, increased CNTF levels in cerebrospinal fluid and/or blood have been described in some human conditions characterized by variably extensive and/or evident tissue damage, including acute disseminated encephalomyelitis^17^, sporadic and familial amyotrophic lateral sclerosis (ALS)^18,19^, focal epilepsy^20^, autism^21^ and septic shock^22^.

The involvement of CNTF in human body metabolism has serendipitously been discovered in clinical trials evaluating its efficacy in ALS patients; although administration of human recombinant CNTF did not ameliorate their motor symptoms, it unexpectedly induced weight loss^23,24^. The weight-reducing effect of Axokine—a recombinant form of human CNTF characterized by greater efficacy and specificity—was later confirmed in a population of leptin-resistant patients with obesity, although the clinical trials were discontinued after a significant number of patients developed autoantibodies^25^. Studies of experimental animals have found that intravenous/intraperitoneal CNTF/Axokine acts on hypothalamic centres to reduce food intake, it induces adipose tissue lipolysis, stimulates brown fat-dependent non-shivering thermogenesis, promotes insulin sensitivity and reduces liver steatosis^26,27^. The metabolic effects of exogenous CNTF rely on the widespread distribution of CNTFRα, which, in striking contrast to its ligand, is widely expressed in metabolically important organs and cell types such as skeletal muscle fibres, white and brown adipocytes, hepatocytes, pancreatic islet cells and intestinal cells^26–28^, [freely available from the GTEx Portal].

Obesity is a chronic disorder associated with complex, interrelated and persistent metabolic dysfunctions, endocrine abnormalities and inflammatory conditions that over time affect and damage specific tissues and organs and impair some cellular functions, eventually leading to obesity-associated diseases such as insulin resistance, type 2 diabetes (T2D), non-alcoholic steatohepatitis, atherosclerosis, cardiovascular disease and even some cancers^29–31^.

In obesity, some types of tissue/cell damage may involve activation of endogenous CNTF signalling which, by virtue of its catabolic action, has the potential to counteract some adverse effects of obesity and of the associated metabolic syndrome. In this observational study, we measured circulating CNTF and CNTFRα in a cohort of patients with obesity with or without diabetes and in age- and gender-matched healthy subjects. Collectively, our findings indicate that circulating CNTF levels are upregulated in such patients and that they correlate with several clinical and/or haematological indices of obesity, inflammation and insulin resistance.

## Materials and methods

### Patients

Blood samples were obtained from 118 patients (median age, 60 years; interquartile range, IQR, 56–65) admitted to the Italian National Research Centre on Aging (INRCA), Ancona, Italy^32^. All subjects provided written informed consent and the original study protocol was approved by the Institutional Review Board of IRCCS INRCA hospital (approval no. 34/CdB/03). The study was carried out in accordance with the Declaration of Helsinki. Participants came from the provinces of Ancona, Ascoli Piceno, Macerata, Fermo and Pesaro-Urbino (central Italy) and provided information such as vital signs, anthropometric measures, medical history and behavioural data including diet and physical activity. All subjects consumed a Mediterranean diet. They were categorized into 3 age- and gender-matched groups (Table 1): control subjects (C; n = 39, 19 men), patients with obesity (O; n = 39, 19 men) and patients with obesity and T2D (OD; n = 40, 20 men). Control subjects came from a larger population enrolled in a T2D prevention programme and were considered healthy because at the time of blood collection they had normal haematological parameters and were free of clinically evident major diseases. Obesity was defined as a body mass index (BMI) ≥ 30 kg/m^2^ according to the World Health Organization classification. T2D was diagnosed according to American Diabetes Association criteria^33^. The presence or absence of T2D complications was established as described previously^34^. Of the 40 patients with obesity and diabetes, 17 had at least one T2D complication and 11 patients had 2 or more; specifically, 8 patients had diabetic neuropathy, 8 had diabetic nephropathy, 6 had diabetic retinopathy, 9 had atherosclerotic vascular disease and 6 had a history of major adverse cardiovascular events. At the time of enrolment, 36 patients with obesity and diabetes were receiving the following medications, alone or in combination: 23, metformin; 15, sulphonylureas; 2, glinides; 5, insulin.

**Table 1:** Clinical characteristics of participants according to their health condition.

Total	Control (n = 39)	Obesity (n = 39)	Obesity and diabetes (n = 40)	*p*	
Gender (M)^#^	19 (48.7%)	19 (48.7%)	19 (47.5%)	0.9922	
Age, y	58 (56; 64.5)	59 (56.5; 63)	63 (56.8; 67)	0.097	
BMI, kg/m^2^	23.7 (22; 24)	32.7 (31.2; 34.8)	34.5 (31.6; 36.8)	< 0.001	(1)
WHR, m	0.9 (0.8; 0.9)	1 (0.9; 1)	1 (0.9; 1)	< 0.001	(1)
Fasting glucose, mg/dL	89 (82; 95.5)	94.5 (92; 103.8)	189.5 (171.8; 230.5)	< 0.001	(2)
Fasting insulin, µU/mL	3.8 (2.4; 5.2)	8.4 (5.6; 9.9)	9.9 (7.8; 11.7)	< 0.001	(1)
HOMA index	0.9 (0.5; 1.1)	1.8 (1.3; 2.5)	4.5 (3.7; 5.3)	< 0.001	(2)
HbA1c, %	5.6 (5.4; 5.9)	5.8 (5.5; 5.9)	8.1 (7.6; 8.8)	< 0.001	(3)
Total cholesterol, mg/dL	218 (200.5; 240.5)	212.5 (185.5; 229.8)	218 (189.8; 235.5)	0.324	
HDL-cholesterol, mg/dL	64 (56.5; 72.5)	52 (45.2; 60.8)	46 (41.8; 49)	< 0.001	(1)
LDL-cholesterol, mg/dL	129.2 (112.2; 148.4)	119.5 (96.6; 141.3)	119.2 (105.3; 144.4)	0.434	
Apolipoprotein A1, mg/dL	192 (164.5; 215)	172 (159; 185.5)	158 (141; 201.2)	0.001	(1)
Apolipoprotein B, mg/dL	104 (87.5; 125.5)	97 (83; 112.5)	109 (98.5; 131.2)	0.072	
Triglycerides, mg/dL	74 (53; 97.5)	104.5 (78.5; 132)	150 (111; 191.8)	< 0.001	(2)
Azotaemia, mg/dL	39 (35.5; 44.5)	39.5 (35; 44.8)	37.5 (33; 46.5)	0.898	
eGFR, mL/min	86.7 (80.4; 100)	83.5 (72.1; 87.5)	80.3 (63.2; 85.2)	0.002	(4)
Creatinine, mg/dL	0.8 (0.7; 0.9)	0.8 (0.7; 1)	0.9 (0.7; 1)	0.006	(4)
Uric acid, mg/dL	4.5 (3.8; 5.4)	5 (4.5; 5.7)	4.9 (4.2; 5.4)	0.040	(5)
Alkaline phosphatase, U/L	69 (59; 81)	68 (58.8; 85)	80 (64.5; 97.5)	0.111	
Aspartate aminotransferase, U/L	21 (17; 23.5)	22 (18.2; 28)	23 (17.8; 26)	0.273	
Alanine aminotransferase, U/L	5 (32; 38)	41.5 (35.2; 49.5)	48.5 (40; 60.2)	< 0.001	(1)
hsCRP, mg/L	0.9 (0.6; 1.6)	2.9 (1.7; 6.6)	5.5 (2.3; 11.6)	< 0.001	(1)
Fibrinogen, mg/dL	268.5 (241.5; 297)	280 (247.5; 297)	331 (280.8; 376)	0.001	(3)
PAI-1, ng/mL	17.6 (12.1; 26.3)	22.2 (16.6; 31.2)	23.7 (17.8; 29.2)	0.072	
IL-6, pg/mL	1.1 (0.8; 1.5)	2 (1.4; 3.1)	2.8 (2.1; 5.1)	< 0.001	(2)
Adiponectin, ng/mL	5718 (4330; 6992)	3282 (2409; 5031)	3020 (2466; 4498)	< 0.001	(1)
Leptin, ng/mL	10.6 (6; 20.1)	34.4 (20; 68.2)	40.3 (28.7; 59.3)	< 0.001	(1)

Values are median and IQR; *p* value refers to Kruskal–Wallis test; ^#^Values are absolute and percent frequencies; *p* value refers to Chi-square test. Multiple comparisons: (1) Obesity, Obesity and Diabetes versus Control; (2) Obesity, Obesity and Diabetes versus Control; Obesity and Diabetes versus Obesity; (3) Obesity and Diabetes versus Control, Obesity; (4) Obesity and Diabetes versus Control; (5) Obesity versus Control.

### Laboratory assays

Peripheral venous blood was drawn from all participants in the morning after overnight fasting. EDTA plasma was obtained within 4 h by centrifugation at 2.000 rpm for 20 min at 4 °C and frozen and stored at − 80 °C until use. The biochemical parameters were assessed by standard procedures. Estimated glomerular filtration rate (eGFR) was calculated according to the CKD-EPI (Chronic Kidney Disease Epidemiology Collaboration) equation based on serum creatinine, age, gender and ethnicity^35^. The homeostasis model assessment (HOMA) index was calculated as fasting insulin (μU/mL) × fasting glucose (mg/dL)/405^36^. The plasma concentrations of CNTF, CNTFRα, leptin, adiponectin, plasminogen activator inhibitor-1 (PAI-1) antigen and IL-6 were assessed using the following ELISA kits according to the manufacturer’s instructions: Human Ciliary Neurotrophic Factor ELISA kit (# CSB-E04527h, Cusabio, Houston, TX, USA); Human CNTFRα ELISA kit (# LS-F49895, LSBio, Seattle, WA, USA); Human Leptin ELISA kit (# EZHL-80SK, Millipore, MO, USA); Human Adiponectin ELISA kit (# AG-45A-0001YEK-KI01, Adipogen Life Sciences, Switzerland); Human PAI-1 ELISA Kit (# RAB0429, Sigma- Aldrich, Missouri, USA); Human IL-6 Quantikine HS ELISA kit (# HS600C, RRID:AB_2893335, R&D System, Minneapolis, MN, USA). All measurements were performed in duplicate. Missing data were as follows: fasting glucose, total cholesterol, HDL-cholesterol, triglycerides, azotaemia, eGFR, creatinine, uric acid, alkaline phosphatase, aspartate aminotransferase and alanine aminotransferase, 1; adiponectin and leptin, 3; IL-6, 6; and fibrinogen, 24.

### Statistical analysis

The power of the study was assessed for plasma CNTF and CNTFRα levels using two-way analysis of variance, a significance level of 5% and the partial omega-squared effect size, which provides a less biased estimate in studies involving a small sample size^37^. The sample size of the study ensured a power greater than 99% to test an effect size of 0.39 for the three-level factor (healthy controls, patients with obesity and patients with obesity and diabetes) and an effect size of 0.27 for the two-level factor (males and females); it had a power of 91% to test an effect size of 0.09 for the interaction between the two factors. The clinical characteristics and plasma parameters of participants were analysed according to their health conditions. Since the quantitative variables did not follow a normal distribution, a non-parametric approach was employed. The quantitative variables were summarized using the median and IQR. Comparisons among conditions were evaluated with the Kruskal–Wallis test. Spearman coefficients and 95% confidence intervals (95% CI) stratified by gender were calculated to estimate the correlation between CNTF and (i) indices of obesity (BMI and waist-to-hip ratio [WHR]); (ii) parameters of glucidic metabolism (fasting glucose, fasting insulin, glycosylated haemoglobin [HbA1c] and HOMA index); (iii) lipid profile (total cholesterol, HDL-cholesterol, LDL-cholesterol, apolipoprotein A1, apolipoprotein B and triglycerides); (iv) kidney function (azotaemia, eGFR, creatinine and uric acid); (v) liver function (alkaline phosphatase, aspartate aminotransferase, alanine aminotransferase); (vi) inflammatory indices (high-sensitivity C-reactive protein [hsCRP] and IL-6); (vii) prothrombotic factors (fibrinogen and PAI-1); (vi) adipokines (leptin and adiponectin); and (viii) plasma CNTFRα levels. The correlation coefficients were considered significant if the 95% CI did not include the zero value. The multiple quantile regression model was applied to evaluate the association of CNTF and CNTFRα, the dependent variables, while health condition and gender were considered as independent variables, by estimating the 50th percentile. The point and 95% CI of regression coefficients for each independent variable and the interaction term were estimated; in the regression model of CNTF, the interaction term between fasting insulin and gender was also included in the analysis. The regression coefficients were considered significant when the 95% CI did not include the zero value. All analyses were performed using R software 4.0.5.

## Results

### Plasma CNTF is increased in men and women with obesity with or without diabetes

**Figure 1:**
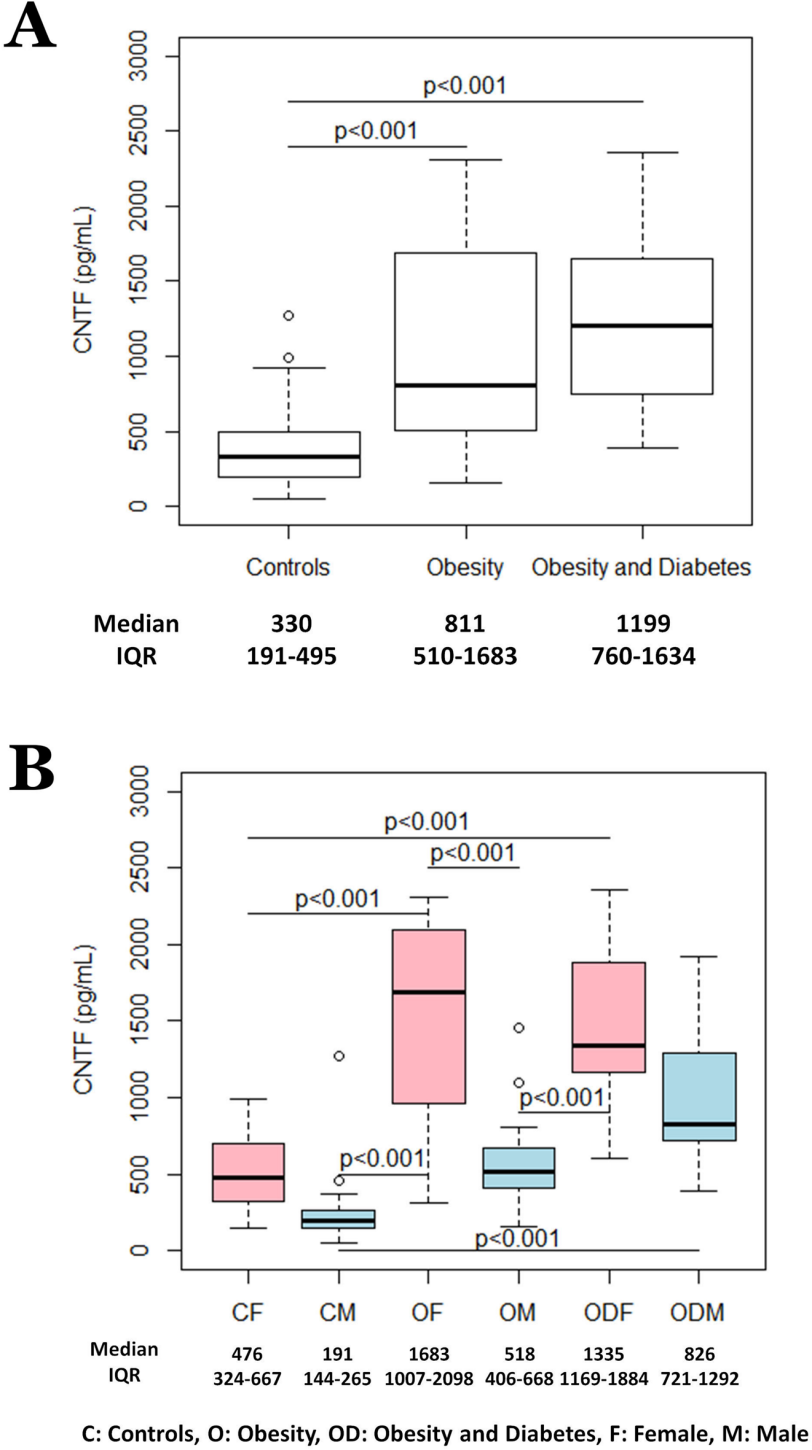
Distribution of plasma CNTF levels according to participants’ health condition (**A**) and health condition and gender (**B**).

As reported in Table 1, the clinical data of participants showed that there were no significant differences in age or gender distribution among the groups. Notably, patients with obesity and patients with obesity and diabetes showed significantly higher BMI, WHR, fasting insulin, hsCRP and leptin and significantly lower HDL-cholesterol and adiponectin levels than Control subjects. Fasting glucose, the HOMA index, triglycerides and IL-6 rose progressively from participants in the Control to Obesity to Obesity and Diabetes cohorts. In the Obesity and Diabetes group, HbA1c and fibrinogen were higher than in the Obesity and Control groups, whereas creatinine was higher and eGFR lower than in Control group. CNTF was detected in the plasma of all subjects. Notably, the two patient groups had significantly higher plasma CNTF than Control subjects; however, although patients with obesity and diabetes exhibited a higher median plasma CNTF level than those with obesity alone, the difference was not statistically significant (Fig. 1A).

Obesity is a sexually dimorphic disease and even its complications have a different incidence and prevalence in men and women^29–31^. Accordingly, plasma CNTF levels were stratified both by clinical condition and gender. Among women, BMI, WHR, fasting insulin, hsCRP, IL-6 and leptin values were higher and adiponectin was lower in the two patient groups than in healthy participants. The HOMA index progressively increased in the three groups. Patients with obesity and diabetes had higher fasting glucose, HbA1c and triglycerides than subjects with obesity or controls, while having significantly lower HDL-cholesterol and eGFR than Control participants (Supplementary Table S1). Among men, the two patient groups had significantly higher BMI, WHR, fasting insulin and leptin and significantly lower HDL-cholesterol levels than Control subjects. In patients with obesity and diabetes, fasting glucose, the HOMA index, HbA1c and IL-6 were significantly higher than in participants in the obesity group and control group. In addition, they showed significantly higher triglycerides, hsCRP and fibrinogen and significantly lower eGFR values than Control subjects. Adiponectin was significantly lower in patients with obesity than in the other two groups (Supplementary Table S2). All female and male patients had significantly higher median values of circulating CNTF than healthy subjects, with marked differences between the genders (Fig. 1B); in particular, unlike women with obesity with or without diabetes, whose CNTF levels were comparable, men with obesity and diabetes showed a higher median CNTF plasma concentration than men with obesity, even though the difference was not statistically significant. In all three groups, circulating CNTF was always higher in women than in men; in particular, the median value was about 2.5-fold higher in Control women, more than 3-fold higher in women with obesity, and about 1.5-fold higher in women with obesity and diabetes. The multiple quantile regression analysis confirmed the independent effect of obesity, alone or associated with diabetes, and gender on plasma CNTF levels; in addition, fasting insulin was positively associated with plasma CNTF, with higher levels in females (Supplementary Table S3).

Collectively, these data show that obesity, with or without diabetes, is associated with increased circulating CNTF levels in both genders. However, whereas in women CNTF secretion was higher in the Obesity group, in men it was higher in group with obesity and diabetes. This suggests that different, gender-related mechanisms underpin its production and secretion in patients with obesity and metabolic syndrome.

### Circulating CNTF correlates with obesity, insulin resistance and inflammation indices

To establish whether circulating CNTF correlates with the major clinical and/or haematological parameters of obesity or metabolic syndrome, we calculated the Spearman correlation coefficient and 95% CI between plasma CNTF concentrations and the parameters listed in Table 1 in each gender, pooling the data of all male and female participants. The values of the Spearman correlation coefficient were graded as follows: 0.20–0.40, weak; 0.41–0.60, moderate; 0.61–0.80 strong; 0.81–1, very strong correlation.

**Figure 2:**
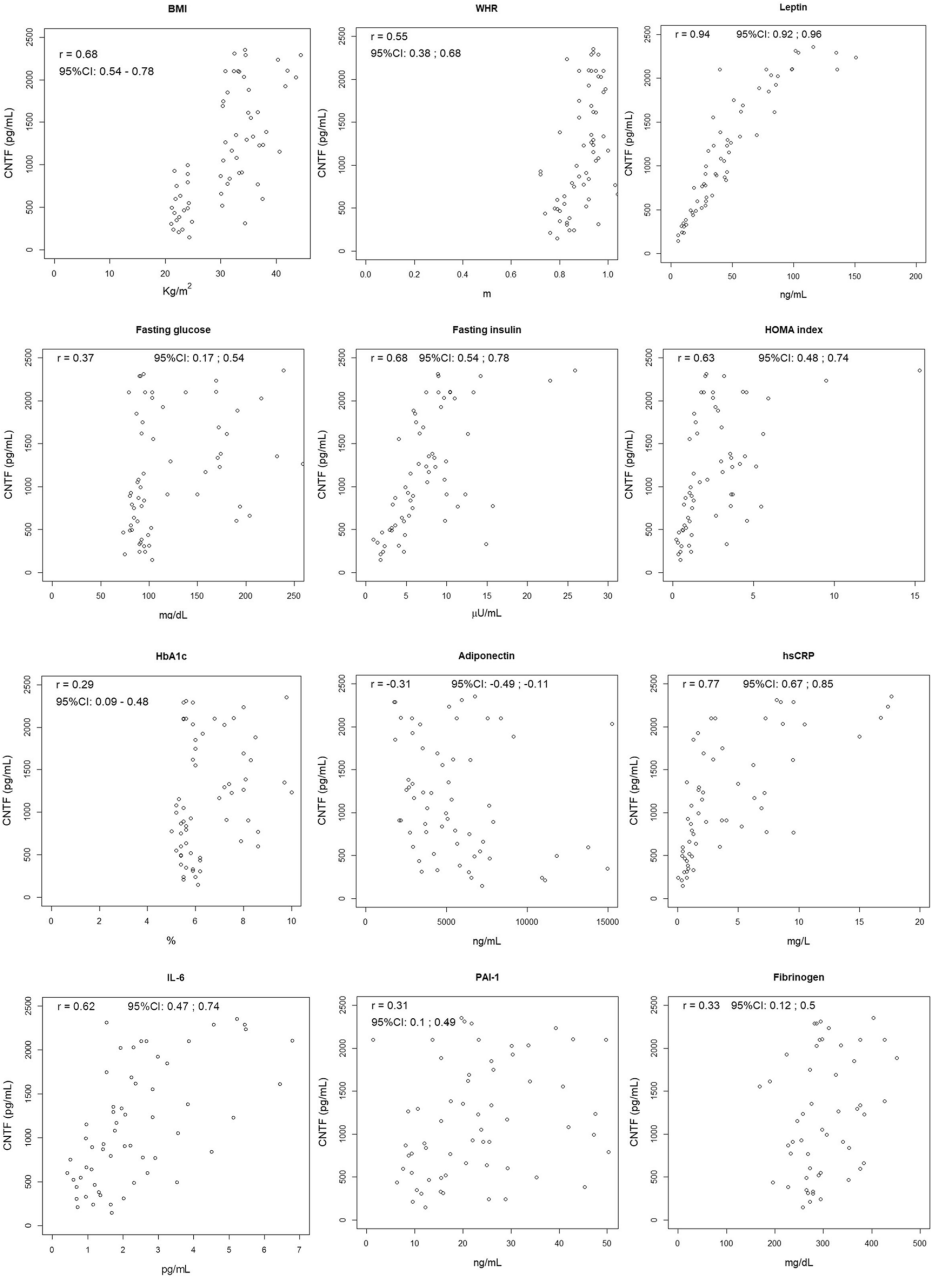
Correlation between plasma CNTF levels and clinical or haematological parameters of obesity, insulin resistance and inflammation in female subjects. Spearman correlation coefficients and 95% CI.

In women (Fig. 2), plasma CNTF showed a moderate correlation with WHR; notably, there was a strong correlation with BMI, the insulin resistance indices fasting insulin and HOMA index and the inflammatory markers hsCRP and IL-6, as well as a very strong correlation with leptin. There was a weak positive correlation with fasting glucose and HbA1c and a weak negative correlation with adiponectin, whose circulating levels are inversely related with body weight, visceral fat accumulation and metabolic syndrome^38^. A weak correlation was also found with the prothrombotic markers PAI-1 and fibrinogen. Finally, plasma CNTF exhibited a moderate correlation with triglycerides and some markers of renal function, including azotaemia and uric acid, and a weak inverse correlation with HDL-cholesterol and eGRF (Supplementary Fig. S1).

**Figure 3:**
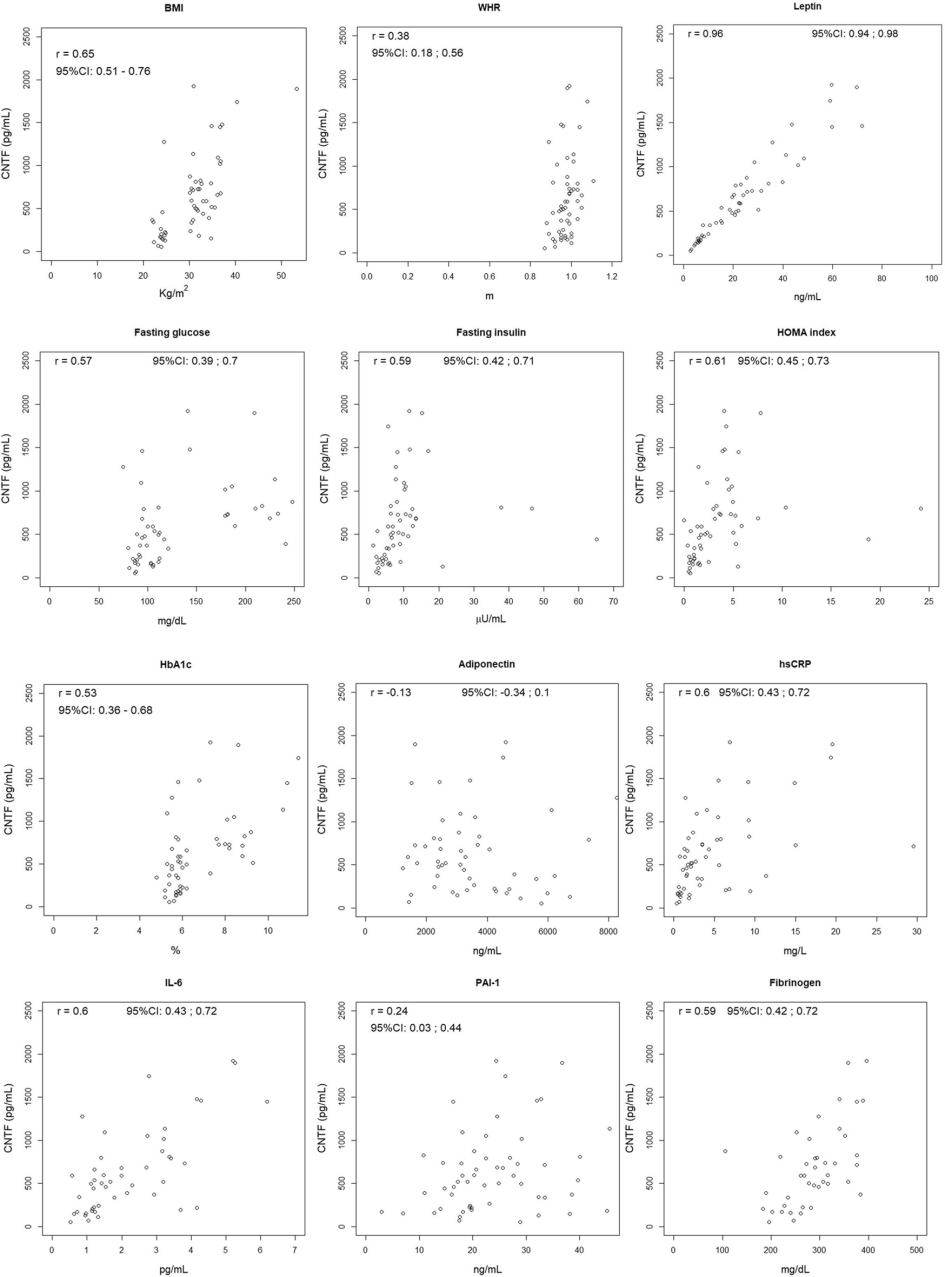
Correlation between plasma CNTF levels and clinical or haematological parameters of obesity, insulin resistance and inflammation in male subjects. Spearman correlation coefficients and 95% CI.

In men (Fig. 3), circulating CNTF showed a weak correlation with WHR; notably, similar to women, there was a strong or very strong correlation with BMI and leptin, respectively. Importantly, there was also a positive correlation with fasting glucose, fasting insulin, HOMA index, HbA1c and the inflammatory markers hsCRP and IL-6. At variance with women, plasma CNTF showed a barely appreciable inverse correlation with adiponectin and a moderate correlation with a single prothrombotic factor, fibrinogen. Finally, there was a weak positive association with triglycerides and creatinine and a weak negative correlation with eGFR and HDL-cholesterol (Supplementary Fig. S2).

### Plasma CNTFRα and the CNTF/CNTFRα molar ratio tend to increase in patients with obesity

**Figure 4:**
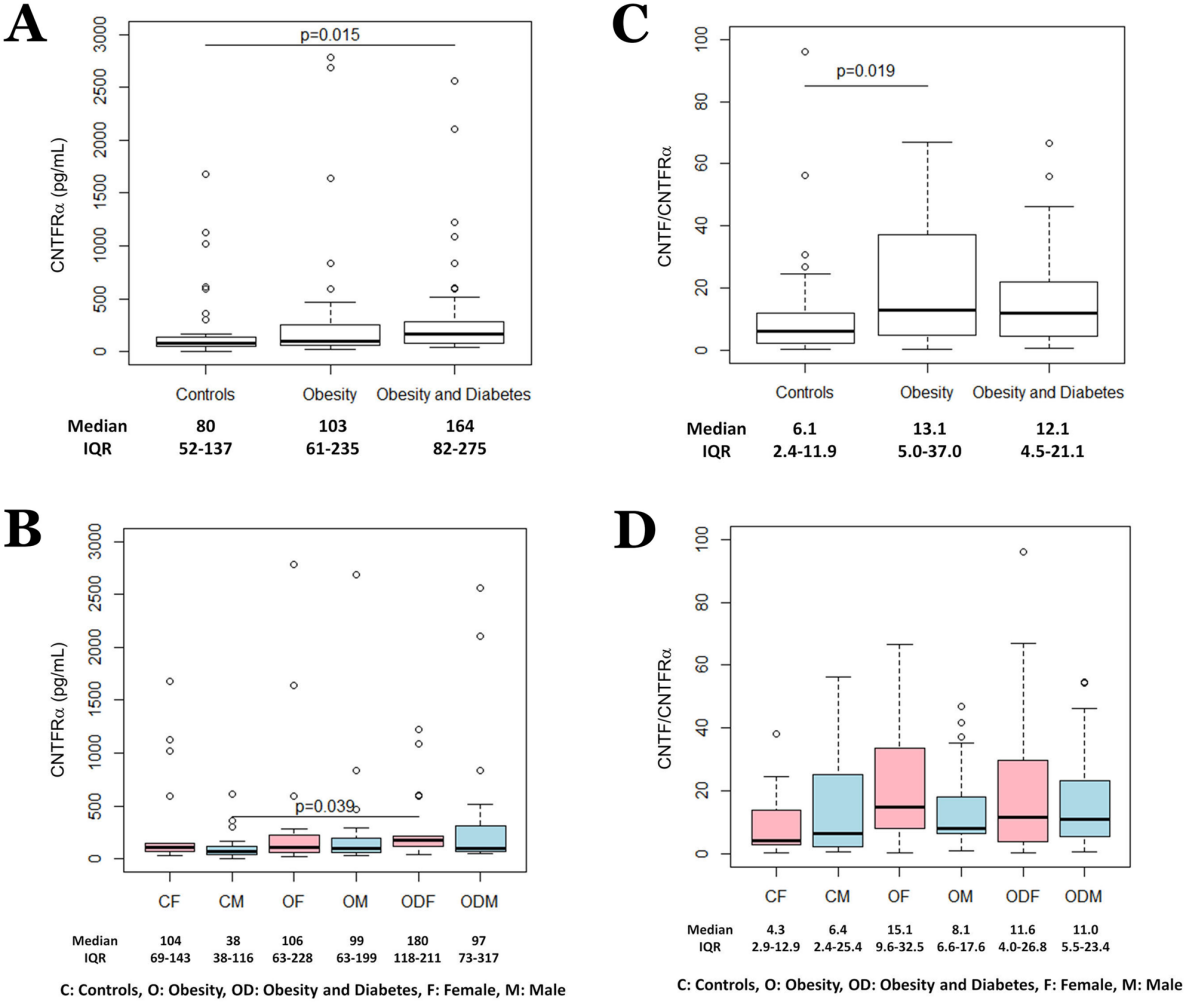
Plasma CNTFRα level distribution according to health condition (**A**) and health condition and gender (**B**). CNTF/CNTFRα molar ratio distribution according to health condition (**C**) and health condition and gender (**D**).

Soluble CNTFRα is detectable in serum and cerebrospinal fluid^39^. Importantly, murine and human cell lines not usually responsive to CNTF become responsive when treated with a combination of CNTF and the soluble form of CNTFRα^39^. These data suggest that, as originally shown for IL-6^40^, systemic CNTF signalling may be sustained, and amplified, by trans-signalling. As a result, the coexistence in the blood of CNTF and its receptor would lead to the formation of a circulating heterodimer that induces responses in discrete target cells originally devoid of the specific cytokine receptor but endowed with LIFRβ and gp130. To determine whether this was the case for CNTF signalling in patients with obesity, we measured plasma CNTFRα and found a progressive increase in median CNTFRα values from the Control group to the Obesity group and the Obesity and Diabetes group, although the difference achieved significance only between patients with obesity and diabetes compared with the Control subjects (Fig. 4A). Gender stratification disclosed lower median CNTFRα concentrations in healthy men and higher CNTFRα levels in women with obesity and diabetes; no further gender-related patterns were evident (Fig. 4B). Interestingly, the presence in each patient group of a substantial number of samples with high/very high CNTFRα levels suggests that in selected patient categories, which were probably underrepresented in our cohort, CNTFRα may be markedly upregulated. Finally, no significant correlation was found between CNTF and CNTFRα plasma levels either in women or in men (data not shown). The results of multiple quantile regression analysis, showing an independent effect of obesity associated with diabetes and gender on plasma CNTF levels, are reported in Supplementary Table S4.

Since the molecular ratio of CNTF-CNTFRα complexes is 1:1^41^, we calculated the CNTF/CNTFRα molar ratio and used it as an index of free circulating CNTF. The ratio was always positive and was higher in patients with obesity with or without diabetes than in healthy subjects, even though the difference was significant only between the Obesity and Control groups (Fig. 4C). Gender stratification confirmed that patients, especially women with obesity, tended to show a higher ratio, but the difference was never significant (Fig. 4D). Collectively, these results demonstrate that human blood contains a higher number of molecules of CNTF than of CNTFRα and that free CNTF tends to increase in patients with obesity.

## Discussion

This cross-sectional study demonstrates for the first time that circulating CNTF values are higher in patients with obesity and in patients with obesity and diabetes than in healthy subjects, and that its plasma concentrations are significantly associated with obesity, diabetes and inflammation indices. Collectively, these findings suggest that in patients with obesity CNTF signalling is activated in a still unknown tissue/organ and that the peptide is released into the circulation, potentially transducing signals to distant organs and affecting body metabolism.

Even in obese patients, where circulating CNTF was highest, its median levels were 2 ng/ml or less. In vitro studies have found variable, often higher, EC50 values of human CNTF ranging from 90 ± 45 pg/ml^42^ to 60 ng/ml^43^, depending on the cell type studied and the biological effects measured. Thus, the increase in circulating CNTF documented in our study could also reflect passive leakage of the peptide into the circulation following CNTF signalling activation in a still uncharacterized site of the body. However, evidence of activation of the specific Janus kinase-signalling transducer and activator of transcription 3 pathway in human adipocytes at a concentration as low as 2 ng/ml^44^ and other possible variables, including linkage with plasma protein and trans-signalling, lend support to the notion that the CNTF oversecreted in patients with morbid obesity may act on discrete cell types at specific sites and play adaptive metabolic roles.

To date, animal studies have failed to provide strong evidence of a role for endogenous CNTF in energy balance control; importantly, neither obesity nor hyperphagia have been described in CNTF-knockout mice^45^, whereas studies of humans with genetic CNTF deficiency do not allow firm conclusions to be drawn^46,47^. However, possible metabolic functions of endogenous CNTF may be revealed under distinctive metabolic challenges that may give rise to tissue and cell damage. Our group has studied CNTF and CNTFRα expression and the distribution of CNTF-producing and CNTF-responsive cells in the hypothalamus of mice rendered obese by a high-fat diet or fed a calorie restriction diet. We found that obesity is associated with increased CNTF hypothalamic signalling, whereas calorie restriction induced its reduction^48^. These data support the hypothesis that, at least at the level of the hypothalamus, obesity can activate endogenous CNTF signalling, which may be involved in energy balance regulation. Notably, aside from the debated role of IL-6 in obesity and insulin resistance, other members of the IL-6 family of cytokines, including CT-1^49^, are oversecreted in obesity and affect the energy balance.

In animal models, it has consistently been demonstrated that CNTF administration not only reduces food intake by acting on hypothalamic^50–52^ and brainstem^53^ feeding centres, but that it also improves obesity-associated hyperglycaemia, hyperinsulinaemia and hyperlipidaemia via metabolic effects exerted through actions on peripheral organs^26,27^. In skeletal muscle, exogenous CNTF increases fatty acid oxidation and reduces insulin resistance^54^; in the liver of *db*/*db* obese mice^55^ and of obese rats fed a high-fat diet^56^, it reduces hepatic steatosis and enhances insulin responsiveness; in mice with alloxan-induced^57^ and streptozotocin-induced^58^ diabetes it protects pancreatic islet cells from cytokine-induced apoptosis, it increases β cell mass and reduces insulin clearance. CNTF also exerts important effects on adipose tissue; in particular, reduces lipogenesis and promotes mitochondrial biogenesis, fatty acid oxidation and insulin sensitivity in white adipocytes^44,59^ and enhances β_3_-adrenergic induction of mitochondrial uncoupling protein 1 in brown adipocytes^60^, possibly promoting thermogenesis-dependent energy expenditure. Although some of these effects may be amplified by the supraphysiological doses of exogenous CNTF used in the experiments, these findings collectively indicate that CNTF exerts central and peripheral anti-obesogenic effects and that in subjects with obesity and diabetes its overproduction could counteract some of the adverse aspects of morbid obesity and metabolic syndrome by reducing energy intake, promoting energy consumption and improving insulin resistance.

The source(s) of endogenous circulating CNTF are unknown. The few cell types where it is known to be constitutively expressed are clearly probable candidates. In patients with obesity, glial cell-derived CNTF may diffuse to the general circulation through the leaking blood–brain barrier^61^, whereas at the periphery metabolic, inflammatory and/or mechanical stress could instigate CNTF secretion by Schwann and/or bone cells. Extensive screening of tissue and cell types from experimental animals challenged with specific metabolic stimuli and selective knockout mouse models are clearly required to identify the cellular source(s) of circulating CNTF.

Interestingly, our data document marked differences between men and women, since the latter show higher constitutive and inducible levels of circulating CNTF. Whereas this finding suggests that sex hormones may instigate differential CNTF production and secretion, the higher amount of this metabolically beneficial cytokine may be involved in the lower and later incidence of some obesity-related complications in female patients^29–31^.

The association between CNTF levels and obesity with or without diabetes was confirmed even when the multiple regression analysis was adjusted for fasting insulin and the interaction between fasting insulin and gender. The positive correlation between fasting insulin levels and body fat mass^62^ suggests the interesting hypothesis that circulating CNTF levels may correlate with adiposity, and that the gender differences observed in our study reflect differences in body fat content between men and women. The accurate measurement of body fat mass by appropriate techniques may enable to establish whether plasma CNTF levels correlate with adiposity and, conceivably, with greater fat accumulation at specific sites (visceral and intra-organ), where it plays a prominent role in the pathophysiology of morbid obesity.

Although human CNTF displays high affinity for CNTFRα, it also binds both membrane-bound and soluble human IL-6 receptor α^63^. Consequently, the high CNTF levels measured in the patient groups may also promote IL-6 signalling, or trans-signalling, compounding the metabolic effects of circulating CNTF. The pleiotropic and redundant functions of IL-6 cytokine family members are also mediated by trans-signalling, as clearly described for IL-6^40^ and IL-11^64^. Trans-signalling occurs when cytokine receptors, secreted or cleaved from the cell membrane, form a circulating complex with the cytokine and extend the signal to a higher number of target cells. CNTFRα lacks a conventional transmembrane domain and is anchored to the cell membrane by a glycosyl-phosphatidylinositol linkage^39^. Thus, specific phospholipases may break down this linkage and elicit the regulated release of the receptor, enabling trans-signalling. In some conditions the circulating heterodimeric complex CNTF-CNTFRα may therefore act on target cells expressing the widely distributed LIFRβ and gp130 proteins, eliciting and/or amplifying responsiveness to circulating CNTF. Even though the higher levels of circulating CNTFRα found in patients with obesity with or without diabetes, agree with the activation of systemic CNTF signalling in obesity, they do not allow conclusions to be drawn with regard to a specific role of CNTF trans-signalling in the pathophysiology of obesity. Finally, the activation of endogenous CNTF signalling in obesity is supported by the higher CNTF/CNTFRα molar ratio found in these subjects, where a larger number of free circulating CNTF molecules are available to signal to CNTFRα-expressing target cells.

In conclusion, human obesity is associated with high plasma CNTF levels, which may have a role in counteracting obesity-induced tissue damage, inflammation and dysmetabolism. Further insights into the metabolic role of endogenous CNTF in homeostatic and pathological conditions and of its sources, target cells and signalling pathways are expected to provide a better understanding of the pathophysiology of metabolic syndrome and improve treatment. Notably, studies involving a higher number of selected patients may disclose circulating CNTF as a potential novel diagnostic and/or prognostic marker of obesity, diabetes and associated diseases.

## Supplementary Information

Supplementary Information.

## Data Availability

The datasets used and/or analysed during the current study are available from the corresponding author on reasonable request.
