# Supplementary material for: Ciliary neurotrophic factor is increased in the plasma of patients with obesity and its levels correlate with diabetes and inflammation indices
Source: Sci Rep. 2022 May 18;12:8331. doi: 10.1038/s41598-022-11942-x (PMC9117681; doi:10.1038/s41598-022-11942-x)
Supplement: Supplementary file 1 — Supplementary Information. [file 41598_2022_11942_MOESM1_ESM.pdf]

Ciliary neurotrophic factor is increased in the plasma of patients with obesity and its levels correlate with diabetes and inflammation indices

Jessica Perugini[1], Eleonora Di Mercurio[1], Angelica Giuliani[2], Jacopo Sabbatinelli[2,3], Anna Rita Bonfigli[4], Elena Tortato[5], Ilenia Severi[1], Saverio Cinti[1,6], Fabiola Olivieri[2,7], Carel W. le Roux[8], Rosaria Gesuita[9] and Antonio Giordano[1,6*]

Supplementary table and figure legends

Supplementary Table S1. Clinical characteristics of female patients according to health condition.

Supplementary Table S2. Clinical characteristics of male patients according to health condition.

Supplementary Table S3. Association of CNTF values with health conditions, gender and fasting insulin. Results from the multiple quantile regression model.

Supplementary Figure S1. Correlation between plasma CNTF levels and clinical or haematological parameters of lipid metabolism and kidney and liver function in female subjects. Spearman correlation coefficients and 95%CI.

Supplementary Figure S2. Correlation between plasma CNTF levels and clinical or haematological parameters of lipid metabolism and kidney and liver function in male subjects. Spearman correlation coefficients and 95%CI.

Supplementary Table S4. Association of CNTFRα values with health condition and gender. Results from the multiple quantile regression model.

Supplementary Table S1. Clinical characteristics of female patients according to health condition.

	Control (n=19)	Obesity (n=19)	Obesity and Diabetes (n=19)	p	
Age, y	58 (54.8 ; 62.8)	59.5 (56.8 ; 65)	62 (55 ; 67)	0.387	
BMI, Kg/m^2	22.6 (21.8 ; 24)	32.7 (31.1 ; 34.7)	35 (33.1 ; 37.5)	<0.001	(1)
WHR, m	0.81 (0.79 ; 0.84)	0.94 (0.91 ; 0.96)	0.94 (0.92 ; 0.97)	<0.001	(1)
Fasting glucose, mg/dL	86 (81 ; 91.25)	93.5 (89.75 ; 100.5)	174 (169 ; 204)	<0.001	(3)
Fasting insulin, µU/mL	3.46 (2.08 ; 4.82)	7.55 (5.5 ; 9.4)	9.8 (7.8 ; 11.4)	<0.001	(1)
HOMA index	0.7 (0.47 ; 1.1)	1.71 (1.29 ; 2.22)	4.14 (3.54 ; 5.16)	<0.001	(2)
HbA1c, %	5.6 (5.4 ; 5.9)	5.6 (5.5 ; 5.9)	8 (7.4 ; 8.5)	<0.001	(3)
Total cholesterol, mg/dL	224 (211 ; 242)	219 (185 ; 233)	214 (194 ; 227)	0.156	
HDL-cholesterol, mg/dL	66 (60 ; 81)	56 (50 ; 64)	48 (43 ; 51)	<0.001	(4)
LDL-cholesterol, mg/dL	130 (117 ; 143)	122 (95 ; 142)	121 (100 ; 146)	0.574	
Apolipoprotein A1, mg/dL	205 (190 ; 218)	179 (161 ; 190)	160 (141 ; 201)	0.003	(1)
Apolipoprotein B, mg/dL	110 (88 ; 124)	102 (78 ; 110)	109 (102 ; 127)	0.206	
Triglycerides, mg/dL	70 (61 ; 99)	99 (77 ; 123)	150 (111 ; 191)	<0.001	(3)
Azotaemia, mg/dL	36 (31 ; 40)	42 (36 ; 46)	36 (33 ; 46)	0.062	
eGFR, mL/min	86 (74 ; 99)	79 (71 ; 86)	83 (63 ; 85)	0.070	(4)
Creatinine, mg/dL	0.7 (0.6 ; 0.8)	0.75 (0.7 ; 0.8)	0.7 (0.7 ; 0.9)	0.127	
Uric acid, mg/dL	3.9 (3.6 ; 4.3)	5 (4.5 ; 5.7)	5.1 (4.2 ; 5.4)	<0.001	(1)
Alkaline phosphatase, U/L	68 (60 ; 89)	71 (54 ; 85)	80 (63 ; 97)	0.339	
Aspartate aminotransferase, U/L	21 (17 ; 23.2)	19.5 (16 ; 22.8)	20 (15 ; 25)	0.847	
Alanine aminostransferase, U/L	34 (32 ; 37)	38 (33 ; 41)	46 (40 ; 60)	<0.001	(3)
hsCRP, mg/L	0.8 (0.4 ; 1.2)	4.5 (1.6 ; 7.3)	5 (2.1 ; 10.5)	<0.001	(1)
Fibrinogen, mg/dL	276 (260 ; 294)	286 (246 ; 298)	328 (274 ; 378)	0.051	
PAI-1, ng/mL	14 (10 ; 26)	21 (15 ; 27)	23 (20 ; 29)	0.181	
IL-6, pg/mL	1.1 (0.8 ; 1.5)	2.5 (1.7 ; 3.1)	2.5 (1.9 ; 4.2)	<0.001	(1)
Adiponectin, ng/mL	6619 (5622 ; 8638)	4458 (3495 ; 5713)	2967 (2653 ; 5108)	<0.001	(1)
Leptin, ng/mL	17.9 (10.7 ; 27.5)	54.3 (42.7 ; 88.9)	46.2 (34.8 ; 71.8)	<0.001	(1)

Values are median and IQR; p-value refers to Kruskal-Wallis test. Multiple comparisons: (1) Obesity, Obesity and Diabetes vs Control; (2) Obesity, Obesity and Diabetes vs Control; Obesity and Diabetes vs Obesity; (3) Obesity and Diabetes vs Control, Obesity; (4) Obesity and Diabetes vs Control; (5) Obesity vs Control.

Supplementary Table S2. Clinical characteristics of male patients according to health condition.

	Control (n=19)	Obesity (n=19)	Obesity and Diabetes (n=19)	p	
Age, y	59 (57 ; 64.5)	59 (56.5 ; 61)	65 (59 ; 66.5)	0.070	
BMI, Kg/m^2	23.9 (22.8 ; 24.4)	32.7 (31.4 ; 34.8)	32.4 (30.8 ; 36.7)	<0.001	(1)
WHR, m	0.92 (0.89 ; 0.96)	0.98 (0.96 ; 0.99)	1.01 (0.98 ; 1.03)	<0.001	(1)
Fasting glucose, mg/dL	89 (87 ; 101)	98.5 (93.25 ; 111)	210 (181.5 ; 237)	<0.001	(3)
Fasting insulin, µU/mL	4.12 (2.82 ; 5.7)	8.94 (5.95 ; 11.8)	10.3 (7.9 ; 11.75)	<0.001	(1)
HOMA index	1 (0.58 ; 1.42)	2.15 (1.48 ; 2.82)	4.86 (4.22 ; 5.4)	<0.001	(3)
HbA1c, %	5.7 (5.4 ; 5.9)	5.8 (5.5 ; 5.9)	8.4 (7.9 ; 9.1)	<0.001	(3)
Total cholesterol, mg/dL	211 (179 ; 234)	206 (188 ; 223)	224 (187 ; 238)	0.662	
HDL-cholesterol, mg/dL	63 (55 ; 68)	49 (42 ; 56)	43 (40 ; 47)	0.002	(1)
LDL-cholesterol, mg/dL	129 (102 ; 155)	116 (99 ; 139)	118 (108 ; 138)	0.771	
Apolipoprotein A1, mg/dL	176 (158 ; 208)	169 (157 ; 177)	157 (142 ; 192)	0.178	
Apolipoprotein B, mg/dL	100 (88 ; 126)	96 (84 ; 117)	113 (91 ; 132)	0.398	
Triglycerides, mg/dL	78 (52 ; 96)	116 (86 ; 135)	151 (126 ; 206)	<0.001	(4)
Azotaemia, mg/dL	43 (40 ; 48)	38 (32 ; 41)	38 (34 ; 46)	0.037	(5)
eGFR, mL/min	97 (84 ; 107)	86 (76 ; 97)	77 (72 ; 85)	0.011	(4)
Creatinine, mg/dL	0.8 (0.75 ; 0.9)	0.9 (0.83 ; 1)	1 (0.9 ; 1.05)	0.008	(4)
Uric acid, mg/dL	5.3 (4.8 ; 5.8)	5.2 (4.6 ; 5.7)	4.8 (4.2 ; 5.3)	0.237	(5)
Alkaline phosphatase, U/L	69 (59 ; 80)	67.5 (63.2 ; 72.2)	74 (65 ; 102)	0.309	
Aspartate aminotransferase, U/L	21 (16.5 ; 23.5)	27 (22.5 ; 29.8)	24 (21 ; 27.5)	0.031	(5)
Alanine aminostransferase, U/L	35 (32 ; 39)	49 (44 ; 55)	51 (44 ; 58)	<0.001	(1)
hsCRP, mg/L	1.4 (0.8 ; 3.1)	2.3 (1.7 ; 4.1)	5.5 (3 ; 12.1)	0.001	(4)
Fibrinogen, mg/dL	256 (217 ; 298)	268 (252 ; 290)	336 (291 ; 358)	0.032	(4)
PAI-1, ng/mL	19 (16 ; 26)	25 (19 ; 33)	24 (18 ; 29)	0.162	
IL-6, pg/mL	1.1 (0.9 ; 1.5)	1.5 (1.2 ; 2.7)	3.2 (2.7 ; 5.2)	<0.001	(3)
Adiponectin, ng/mL	4407 (3358 ; 5610)	2622 (2362 ; 3184)	3074 (1962 ; 3735)	0.014	(5)
Leptin, ng/mL	6.1 (4.4 ; 7.8)	20.3 (15.4 ; 22.9)	30.7 (25.5 ; 45.4)	<0.001	(1)

Values are median and IQR; p-value refers to Kruskal-Wallis test. Multiple comparisons: (1) Obesity, Obesity and Diabetes vs Control; (2) Obesity, Obesity and Diabetes vs Control; Obesity and Diabetes vs Obesity; (3) Obesity and Diabetes vs Control, Obesity; (4) Obesity and Diabetes vs Control; (5) Obesity vs Control.

Supplementary Table S3. Association of CNTF values with health conditions, gender and fasting insulin**.** Results from the multiple quantile regression model.

	Regression coefficients	95%CI
Intercept	289	210 ; 328
Males vs Females	-92	-155 ; -32
Obesity vs Control	997	460 ; 1159
Obesity and Diabetes vs Control	453	291 ; 637
Fasting insulin (µU/mL)	66	57 ; 107
Obesity in Males	-665	-785 ; -451
Diabetes and Obesity in Males	210	-63 ; 536
Fasting insulin (µU/mL) in Males	-67	-94 ; -57

Supplementary Figure S1. Correlation between plasma CNTF levels and clinical or haematological parameters of lipid metabolism and kidney and liver function in female subjects. Spearman correlation coefficients and 95%CI.

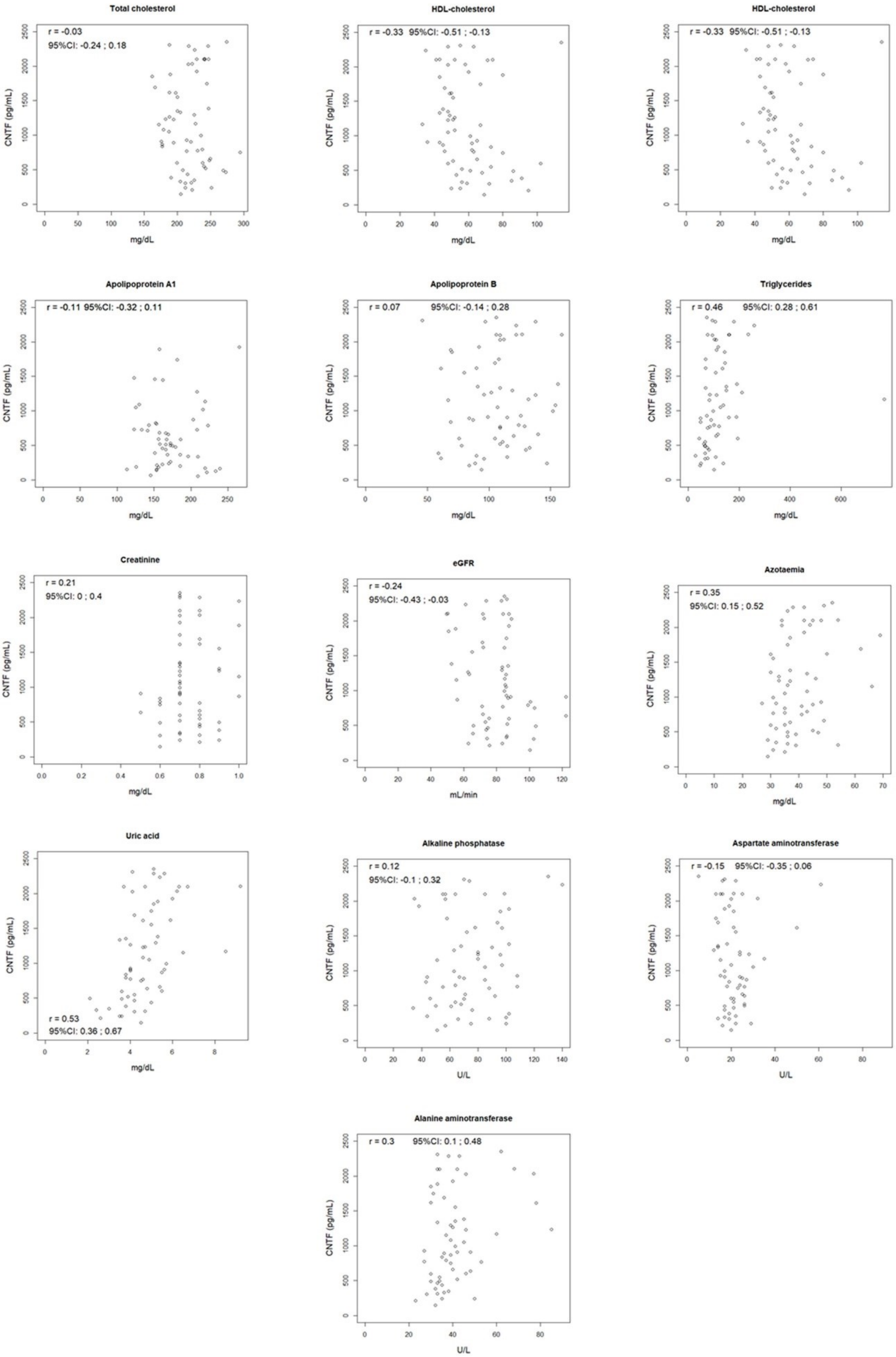

Supplementary Figure S2. Correlation between plasma CNTF levels and clinical or haematological parameters of lipid metabolism and kidney and liver function in male subjects. Spearman correlation coefficients and 95%CI.

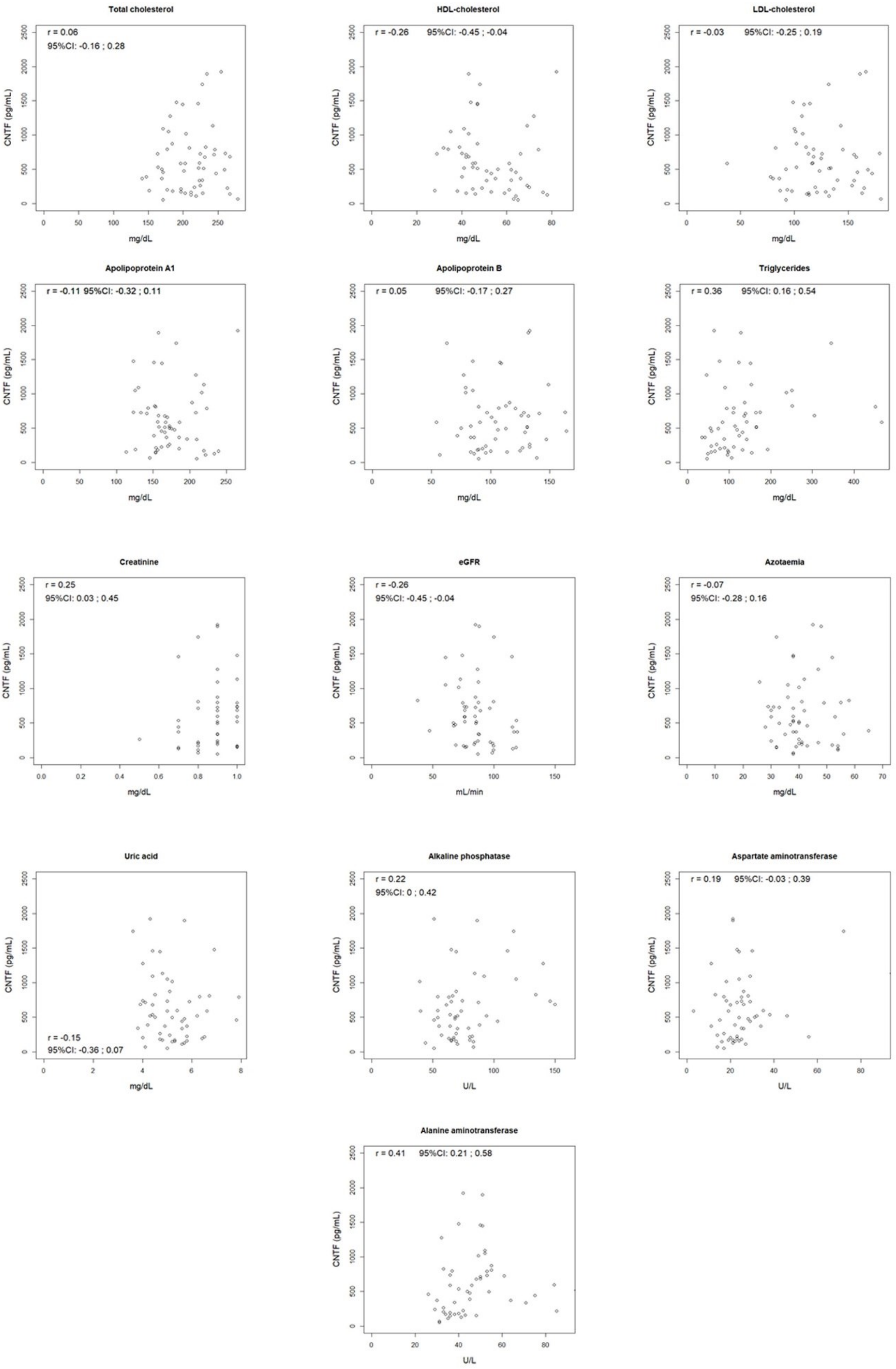

Supplementary Table S4. Association of CNTFRα values with health conditions and gender. Results from the multiple quantile regression model.

	Regression coefficients	95%CI
Intercept	104	79; 125
Males vs Females	-35	-56; -15
Obesity vs Control	1.24	-26; 37
Obesity and Diabetes vs Control	76	24; 109
Obesity in Males	29	-15; 80
Diabetes and Obesity in Males	-48	-96; 106
